# Thymoquinone-loaded mesenchymal stem cell-derived exosome as an efficient nano-system against breast cancer cells

**DOI:** 10.22038/IJBMS.2022.64092.14116

**Published:** 2022-06

**Authors:** Mahboubeh Ebrahimian, Maryam Hashemi, Leila Etemad, Zahra Salmasi

**Affiliations:** 1 Nanotechnology Research Center, Pharmaceutical Technology Institute, Mashhad University of Medical Sciences, Mashhad, Iran; 2 Department of Pharmaceutical Biotechnology, School of Pharmacy, Mashhad University of Medical Sciences, Mashhad, Iran; 3 Pharmaceutical Research Center, Pharmaceutical Technology Institute, Mashhad University of Medical Sciences, Mashhad, Iran; 4 Department of Pharmaceutical Nanotechnology, School of Pharmacy, Mashhad University of Medical Sciences, Mashhad, Iran

**Keywords:** Adipose-derived, mesenchymal, stem cells, Breast cancer, Drug delivery system, Exosome, Thymoquinone

## Abstract

**Objective(s):**

Exosomes became the subject of extensive research in drug delivery approach due to their potential applicability as therapeutic tools for cancer therapy. Thymoquinone (Tq) is an anti-cancer agent due to its great anti-proliferative effect. However, poor solubility and weak bioavailability restrict its therapeutic applications. In this study, exosomes secreted from human adipocyte-derived mesenchymal stem cells (AdMSCs) were isolated and the efficacy of a novel encapsulation method for loading of Tq was investigated. Finally, the cytotoxic effect of Tq incorporated exosomes against cancer cells was evaluated.

**Materials and Methods:**

Exosomes secreted from AdMSCs were isolated via ultracentrifugation and characterized by electron microscopy and western blotting. Then, through a novel encapsulation approach, Tq was loaded into exosomes by the combination of three methods including incubation, freeze-thawing, and surfactant treatment. Then, the encapsulation efficiency, in vitro cellular uptake, and cytotoxicity of Tq incorporated exosomes (Tq@EXOs) in MCF7 and L929 cells were estimated.

**Results:**

Tq loading into exosomes through our novel method caused a significant improvement in encapsulation efficiency of about 60%. The fluorescent microscopy and flow cytometry outcomes indicated the efficient uptake of Tq@EXOs-FITC by cells throughout 4 hr. Furthermore, MTT results displayed the ability of Tq@EXOs in effectively decreasing the cell viability of MCF7 without causing any obvious cytotoxicity on L929 as normal cells.

**Conclusion:**

The results suggest that our approach provides effective loading of Tq into exosomes which offer a valuable and safe platform for drug delivery to cancer cells thus having a great potential for clinical studies.

## Introduction

Exosomes are small extracellular vesicles (EVs) that function as essential intercellular communicators and play an important role in different physiological and pathological mechanisms (1, 2). Exosomes (EXOs) with a diameter range of ~ 40–200 nm are secreted by various kinds of cells including dendritic cells (3), macrophages (4), B cells (5), T cells (6), mesenchymal stem cells (7), endothelial progenitor cells (8), epithelial cells (9), and a variety of cancer cells (10). The great potential of this substance for performing drug delivery arises from varying factors including their natural origin, the ability to cross over different barriers in the body such as the blood-brain barrier (BBB) (11), being non-immunogenic (12), less toxicity than artificial nanoparticles (13), offering high flexibility and compatibility in different administration routes, (14) and having various loading approaches such as physical, chemical, and biological methods (15). Recently, different types of biomolecules and chemical drugs were favorably loaded into exosomes for being applied in cancer treatments. In comparison to free doxorubicin, the loading of doxorubicin into dendritic cells derived targeted exosomes could significantly inhibit the growth of breast cancer cells (16). In addition, reports indicated that paclitaxel-loaded macrophage-derived exosomes could efficiently inhibit the proliferation of Lewis Lung Carcinoma cells when compared with solitary paclitaxel (17). In another case, pancreatic cancer cells derived exosomes that were encapsulated with hydrophobic curcumin caused remarkable cell apoptosis in PANC‐1 and MIA PaCa‐2 cells (18). According to growing studies, the RNAs and proteins of MSC-derived exosomes can affect the fate of tumor cells. The derived exosomes from human MSCs can be suggested as suitable candidates for drug delivery due to their cell-specific tropism and proper biocompatibility (19). 

In recent years, the varying anti-cancer features of herbal compounds along with their low level of cytotoxicity were approved. Thymoquinone (Tq) is one of the bioactive compounds of black seed (*Nigella sativa*) that has been employed as an anti-oxidant, antineoplastic, anti-inflammatory, and analgesic agent (20). The anticancer effect of this substance functions through different mechanisms including the initiation of apoptosis and inhibition of cell proliferation, while there are reports on the involvement of Tq in other mechanisms related to tumor formation such as cell migration, invasion, and alteration of the epigenetic phenomenon in cancerous cells (21). However, poor solubility, weak bioavailability, and fast biotransformation resulted in restricting its therapeutic applications (22). Although nanotechnology succeeded in improving the bioavailability of therapeutic compounds, nevertheless, the rapid clearance of most nano-formulations by mononuclear phagocytic systems (MPS) and their toxic effects stand as considerable obstacles in their applications as drug delivery systems (23). In this regard, exosomes with low cytotoxicity could be suggested as an efficient nano-delivery system for avoiding rapid clearance and overcoming biological barriers (24).

In the present study, we extracted exosomes, which were shed from adipocyte-derived mesenchymal stem cells (AdMSCs), and evaluated their physicochemical properties. Subsequently, Tq was loaded into exosomes through a novel technique based on the combination of three methods including incubation, freeze-thawing, and surfactant treatment. To the best of our knowledge, this is the first report in which a combination of three methods was used for loading therapeutic agents to exosomes. Furthermore, evaluation data was gathered on encapsulation efficiency, cellular uptake, and cytotoxicity of Tq-loaded exosomes in MCF7 and L929 cells. 

## Materials and Methods

***Materials***

Fetal bovine serum (FBS) and exosome-depleted FBS were purchased from Gibco BioCult (Paisley, UK). APC anti-human CD45, FITC anti-human CD34, anti-human CD90, and PE anti-human CD11b and CD73 were procured from BD‐Pharmingen. In addition, anti- CD63, anti-CD9, anti-CD 81, and anti-Bcl2 antibodies were purchased from Abcam (Cambridge, MA, USA). Fluorescein isothiocyanate (FITC), Thymoquinone (≥ 98%), and (4, 5-imethylthiazol- 2-yl)-2, 5-diphenyltetrazolium bromide (MTT) were obtained from Sigma–Aldrich Co (St Louis, MO, USA). 

***Adipose tissues collection***

In order to perform the liposuction technique, adipose tissue samples were collected from healthy donors subsequent to documenting the consent signature of each patient. All of the involved processes were approved by the Medical Sciences Review Committee (Approval number IR.MUMS.PHARMACY.REC.1397.026) of Mashhad University of Medical Sciences. Adipose samples were stored in Dulbecco’s Modified Eagle Medium (DMEM) with 1% (v/v) penicillin/streptomycin to be instantly shipped to the laboratory (25). 

***Isolation and culture of*** ***adipose*** ***‐derived mesenchymal stem cells (AdMSCs)***

Adipose sample (50 ml) was completely washed with PBS that contained 1% (v/v) penicillin/streptomycin to be mixed and incubated with collagenase type I solution (0.1%) for about 40 min at 37 °C within a shaker incubator. Once the collagenase was neutralized through the addition of a culture medium composed of fetal bovine serum (FBS) (10% v/v), the cell suspension was filtered and centrifuged at 1500 rpm for 10 min. Subsequently, the obtained pellet was resuspended in a culture medium and the resulting cells were kept at 37 °C in a humidified incubator that contained 5% CO₂ (26). 

***AdMSCs characterization***

***Immunophenotyping of AdMSCs***

The expression of cell surface markers was evaluated to confirm the identity of AdMSCs. For this purpose, the cells were detached from cell culture flasks and incubated with APC anti-human CD45, FITC anti-human CD34 and CD90, PE anti-human CD11b, and CD73 antibodies at 4 °C for 20 min. Then, the cells were washed with PBS and analyzed by the means of flow cytometry (FACS Calibur machines; Becton Dickinson) (27). 

***AdMSCs differentiation***

*Adipogenic differentiation*

Subsequent to 3 passages, AdMSCs were cultured in a 12-well plate with the density of 4 × 10^4 ^cells/well. Then, dexamethasone (10^-6 ^M), insulin (10 µg/ml), and indomethacin (100 µM) were added to the culture medium that was supplemented with 10% FBS and 1% antibiotic solution for adipogenic induction. The media was exchanged with fresh induction culture medium every three days for a time interval of 21 days. As the last step, the cells were stained with Oil Red O (28, 29). 

*Osteogenic differentiation*

AdMSCs were cultured for 21 days under osteogenic induction culture media with the composition of dexamethasone (10-7 M), β-glycerophosphate (10 mM), and L-ascorbic acid 2-phosphate (0.2 mM). The medium was exchanged every three days. Finally, osteocytes were fixed to be stained with Alizarin Red S (30). 

***Exosome isolation from AdMSCs***

AdMSCs were cultured with L-DMEM that was supplemented with FBS 10% (v/v). Subsequent to 3–5 passages, once AdMSCs reached the confluency of about 70%, they were washed three times with PBS to be incubated with L-DMEM medium that was enriched with commercial exosome-depleted FBS (10%). Subsequently, supernatants were collected from AdMSCs conditioned medium after 72 hr to perform the exosome extraction. Then, serial centrifugations were conducted to eliminate all of the intact cells, dead cells, cell debris, and larger particles. To do so, the conditioned medium was centrifuged at 300 × g for 10 min, 1000 × g for 20 min, and 10,000 × g for 30 min at 4 °C. Thereafter, the supernatant was filtered through a 0.2 µM filter to remove the remaining large particles, which was followed by ultracentrifugation (Hitachi, Himac, CS150GXL, Hokkaido, Japan) at 120,000 × g for 70 min at 4 °C. The obtained pellet was washed with cold phosphate-buffered saline (PBS) to be ultracentrifuged once again at similar conditions (120,000 × g, 70 min). The pelleted exosomes were kept at −80 °C after resuspension in PBS (31). 

***Protein quantification in extracted exosome***

The resulting pellet from ultracentrifugation was resuspended in PBS. The protein content of extracted exosomes was measured by the application of a BCA protein assay kit (Parstous, Iran), which was performed in accordance with the manufacturer’s instructions. Their related absorbance was also measured at 562 nm through application of Infinite® 200 PRO multimode microplate reader (Tecan Group Ltd. Männedorf, Switzerland).

***Exosome characterization***

*Scanning electron microscopy (SEM) and particle size analysis*

Field emission scanning electron microscopy (FE-SEM, Mira IIIFEG, TESCAN-UK, Ltd) was utilized to check the morphology of our exosomes. For this purpose, the exosome solution in PBS was placed on a metal stub and dried under the hood at room temperature (RT) to be prepared for FE-SEM imaging. The hydrodynamic diameter and zeta potential of exosomes were examined by the means of DLS on a Zetasizer Nano ZS (Malvern Instruments, Malvern, UK). The exosomes (1 µg) were diluted in PBS (1 ml) and the data of three independent measurements were gathered.

*Western blot analysis*

Western blot analysis was performed according to the previous description along with some modifications (32, 33). Briefly, exosomes were lysed by the usage of lysis buffer containing (750 mM of NaCl, Triton X- 100 5.0%, 250 mM of Tris-Cl, pH=8.0) with fresh addition of proteinase inhibitor cocktails (Kiazist, Iran). Then, the lysates were removed by centrifugation at 14,000 rpm at 4 °C for 20 min. In order to determine the protein concentration of exosome lysates, we used a BCA protein quantification kit according to the manufacturer’s instructions. Next, exosome lysates were mixed with equal volumes of 2X Laemmli sample buffer to be boiled for 5 min. Lysates (15 μg) were then subjected to SDS-PAGE and subsequently transferred to a 0.2 μm Immune-Blot™ polyvinylidene difluoride (PVDF) membrane (Cat No: 162-017777; Bio-Rad Laboratories, CA, USA). Thereafter, the membranes were blocked with 5% BSA (Cat No: A-7888; Sigma Aldrich, MO, USA) in 0.1% Tween 20 for 1 hr. The next step required the incubation of membranes with anti-CD63, anti- CD9, anti-CD81, and anti- Bcl-2 antibodies for 1 hr at room temperature. Subsequently, membranes were washed three times with TBST to be incubated with goat anti-rabbit IgG H&L (HRP) (Cat No: ab6721; Abcam) as the secondary antibody. The membranes were then visualized through an enhanced chemiluminescence substrate (ECL). 

***Tq loading into exosome (Tq@exo)***

Tq solution (152 µM) was mixed with different amounts of isolated exosomes (100, 200, and 400 µg) in a culture medium. For approaching Tq incorporation with high efficiency into exosomes, we used our novel method that is based on combination of three conventional techniques including simple incubation, surfactant method, and freeze-thawing cycle. Initially, Tq was incubated with different concentrations of exosomes on a rotary shaker by addition of 0.1% tween-20 for 18 hr at room temperature. Then, the solution was instantly freeze-thawed three times at – 80 °C and 37 °C. To determine the efficiency of Tq loading, the solution was ultracentrifuged for 70 min at 120,000 ×g and the absorbance of the supernatant was measured at 270 nm. Subsequently, Tq loading efficiency was evaluated through an indirect method and calculated by the following equation: 

$\mathrm{Amount of EE}\left(\%\right)=\frac{\mathrm{Total amount of used Tq}-\mathrm{Amount of inloaded Tq}}{\mathrm{Amount of used Tq}}\times 100$

Tq-loaded exosomes were collected and washed with PBS three times and the pellet was resuspended in a minimum amount of PBS and kept at -80 °C for the next experiments (34). 

***Cell viability assay***

At first, we investigated the cytotoxicity of free Tq on MCF7 and L929 cells by the usage of an MTT assay. The cells were seeded in a 96-well plate for 24 hr prior to the treatment. Then, we added a fresh complete medium (100 µl) that contained Tq in a concentration range of 9.5 to 304 µM to each well, while repeating each concentration in triplicate. After 24 hr and 72 hr, MTT reagent (20 µl, 0.5 mg/ml PBS) was added to each well to be incubated for 4 hr at 37 °C. Then, dimethyl sulfoxide (DMSO, 100 μl/well) was added after removal of the culture medium in order to dissolve the formazan crystals by the usage of a plate shaker (Behdad, Iran) at 300 rpm for 20 min. The absorbance was read at the wavelengths of 570 and 630 through a microplate reader (Infinite NanoQuant M200, Tecan, Switzerland), while the untreated cells were considered as control (35, 36).

Once the IC_50_ of free Tq was determined, we evaluated the cytotoxicity of Tq@exos in MCF7 and L929 cells in concentrations lower than the IC_50_ of Tq. 

***Cellular internalization of exosomes***

The staining process of the Tq@exo membrane with FITC was conducted in accordance with the previous protocol (37). Briefly, FITC (0.25 µg/µl) was added drop wisely to the Tq@exo solution, which was being gently stirred, and incubated in darkness for 2 hr at room temperature. Unbounded FITC was removed through two cycles of ultracentrifugation at 120,000 × g for 70 min while exosomes-free FITC dye was exerted as the background control. Next, MCF7 cells were incubated with the cases of Tq@exo-FITC and solitary FITC dye for about 4 hr at 37 °C, which were then analyzed qualitatively using a JuLI Smart fluorescent cell analyzer (MA, USA) and quantitatively by flow cytometry. 

***Statistical analysis***

The results were expressed as mean±standard deviation (mean±SD) with at least 3 replications. Statistical analysis was performed in Prism 6 software (Graphpad, La Jolla, CA, USA) through application of one-way Analysis of Variance (ANOVA), which was followed by Dunnett’s multiple comparisons test. *P*-values<0.05 were considered to be statistically significant and equivalent to *, while *P*<0.01 and *P*<0.001 were considered to be equivalent to ** and ***, respectively.

## Results

***Characterization of isolated AdMSCs***

*Flow cytometry analysis*

**Figure 1:**
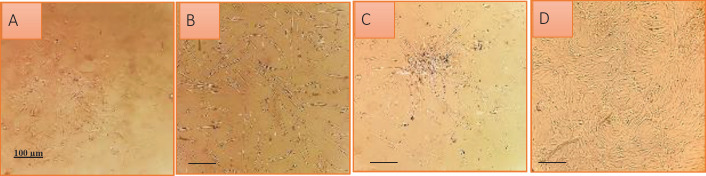
Morphologic characterization of Adipocyte‐derived mesenchymal stem cells (AdMSCs). (A) The spindle shape of AdMSCs appeared at third day. (B-D) Homogenous population of cells developed with suitable confluency after three passage

**Figure 2:**
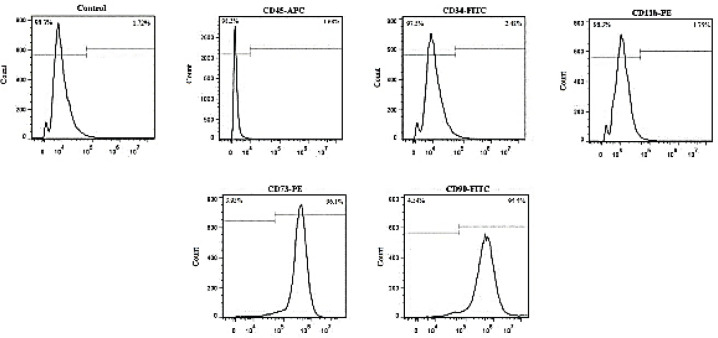
Expression of cell surface antigens were confirmed in isolated AdMSCs using flowcytometry. Cells were positive for CD73 and CD90 which are popular for human adipocyte-derived mesenchymal stem cell markers and negative for CD45, CD34 and CD11b which are hematopoietic stem cell markers

Isolated AdMSCs were characterized in terms of morphology (Figure 1) and cell surface antigens at passage 3. The obtained results indicated that the cells were positive for human AdMSC markers (CD90 and CD73) and observed to be negative for hematopoietic stem cell markers (CD45, CD34, and CD11b) (Figure 2). 

*AdMSCs differentiation*

**Figure 3:**
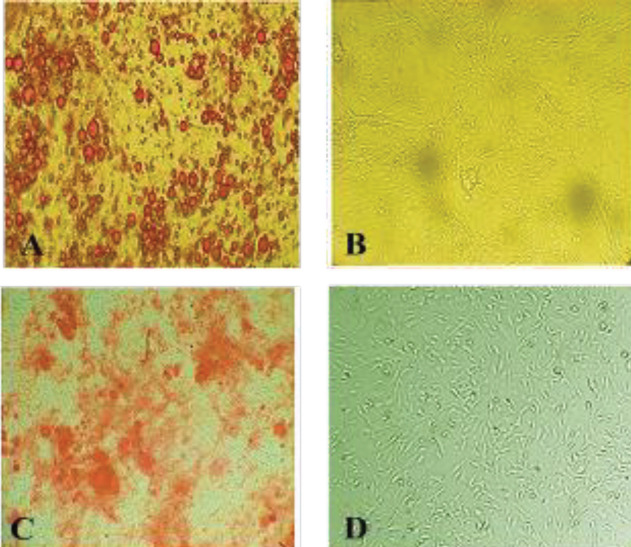
Multilineage differentiation ability of AdMSCs. (A) Oil Red O staining of AdMSCs differentiated into adipocytes containing oil droplets. (B) Untreated cells as the control

The differentiation potential of AdMSCs was inspected for osteoblast and adipocyte lineages. In this regard, AdMSCs were cultivated in osteogenic and adipogenic induction mediums for 21 days. As displayed in Figure 3A, the outcome of Oil Red O staining confirmed the accumulation of intracellular lipid droplets after three weeks. Furthermore, calcium deposits were determined within the cytoplasm through the Alizarin Red S staining (Figure 3B); untreated AdMSCs were regarded as the control. 

***Exosome isolation and characterization***

**Scheme 1:**
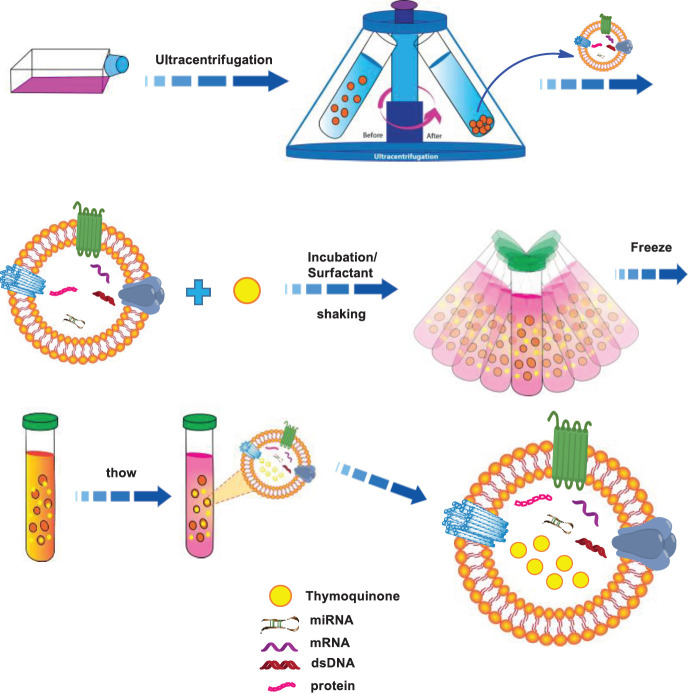
Schematic illustration of Tq loaded into EXOs using a novel technique based on the combination of three methods: incubation, freeze-thawing and surfactant treatment

In the present study, Tq was loaded into EXOs through our novel approach as it is displayed in Scheme 1.

*Evaluation of protein content in extracted exosomes*

The protein concentration of extracted exosomes was evaluated by application of bicinchoninic acid (BCA) assay and calculated based on the standard BCA curve. According to the results, the protein content of exosome, extracted from 10 × 10 ^6 ^AdMSCs, was observed to be 260 µg.

*FE-SEM analysis*

**Figure 4.**
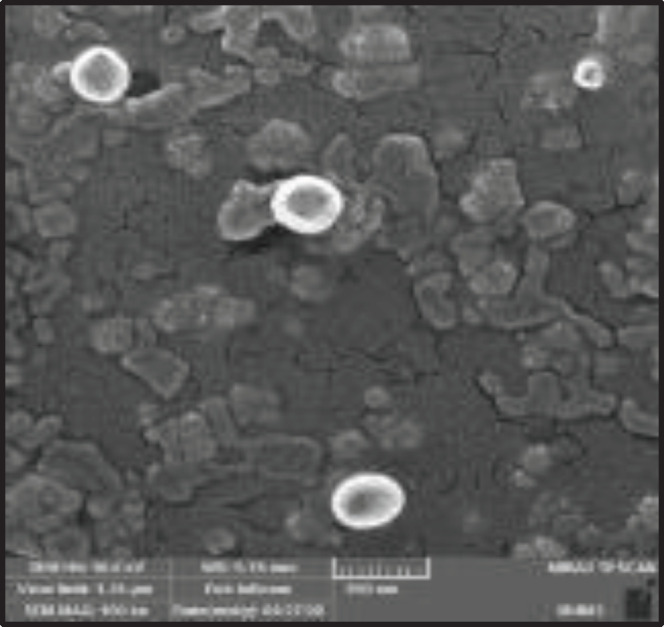
Field emission scanning electron microscopy (FE-SEM) image of exosomes

The FE-SEM analysis was considered to assess the morphology of extracted exosomes, which were detected to be homogenous with a spherical shape as presented in Figure 4. The outcome of DLS analysis revealed the size of exosomes to be 110 nm along with a zeta potential of about -21.3 mV.

*Western blot analysis*

**Figure 5:**
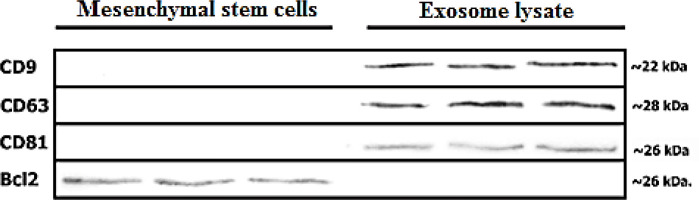
Western blot analysis of CD9, CD63 and CD81 as positive marker and Bcl2 as negative marker of isolated exosomes

The results of investigating the expression of exosome-related markers by application of western blot experiment confirmed the presence of CD81, CD63, and CD9 (as tetraspanin proteins) on the exosomes. Furthermore, we detected the expression of Bcl2 (an intracellular protein marker in the role of a negative marker) throughout the MSCs while being absent in the exosomes (Figure 5). 

***Loading of Tq***

Tq was loaded into exosomes via an innovative method that involves the combination of three conventional techniques including simple incubation, surfactant method, and freeze-thawing cycle. Subsequent to the ultracentrifugation of Tq@EXOs, the statues of free Tq in the supernatant were evaluated by the usage of UV–Vis spectrophotometry at 270 nm. The best result was obtained from the Tq/exosome ratio of 1/16 with the encapsulation efficiency percentage of 57 + 3. The results of each method that was performed in this study for loading Tq to exosomes are listed in Table 1.

***MTT assay***

**Figure 6:**
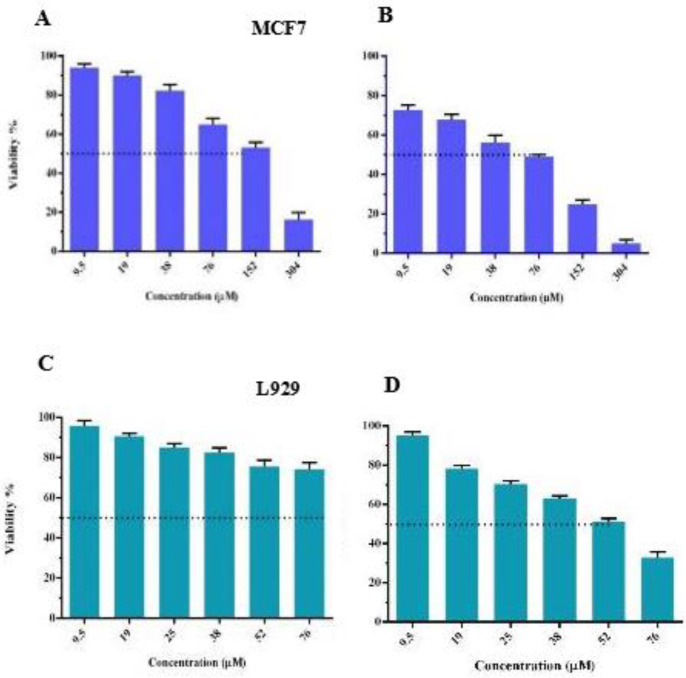
The cytotoxic activity of free Tq against MCF7 and L929 cell line after 24 hr (A and C) and 72 hr (B and D). IC_50_ of free Tq in MCF7 and L929 cells after 72 hr was obtained 76 µM and 52 µM, respectively. The experiment was done in triplicate for different time point

MTT assay was performed to study cell viability by utilizing different concentrations of free Tq at the varying time intervals of 24 and 72 hr. The obtained results indicated that the IC_50_ of free Tq in MCF7 cells after 24 and 72 hr were 152 and 76 µM, respectively. Meanwhile, the IC_50_ of free Tq in L929 cells was obtained to be 52 µM after 72 hr (Figures 6A-D). 

**Figure 7:**
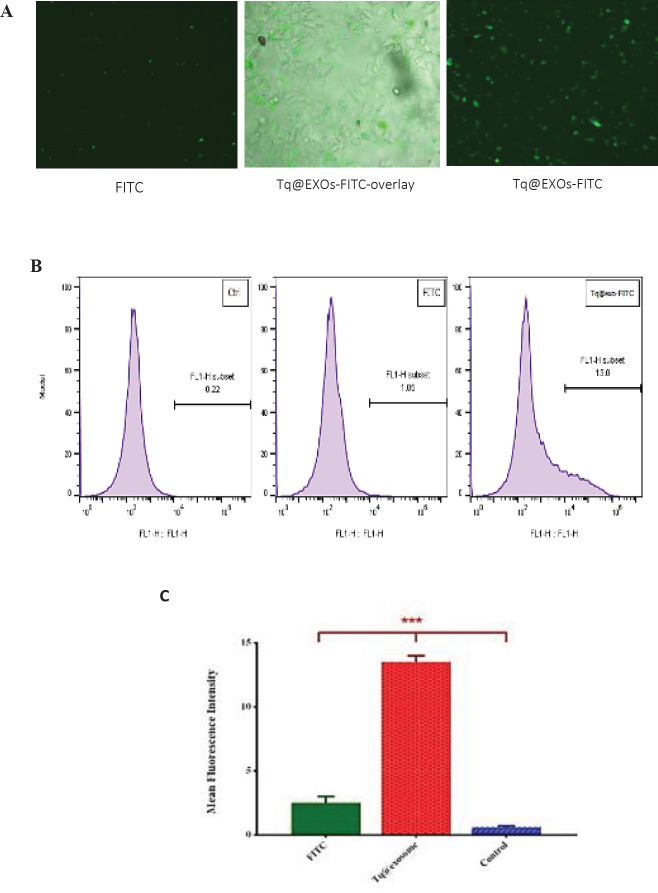
Cellular toxicity of Tq@EXOs in comparison with free Tq after 72 hr, in concentrations range of IC_50_, IC_25_ and IC_10_ in L929 and MCF7 cells. Tq@EXOs could effectively decrease the MCF7 viability in all IC concentrations compare to L929. Mean± SD of three independent experiments using GraphPad Prism 6

Next, MCF7 and L929 cells were treated with free Tq and an equivalent dosage of Tq-loaded EXOs for 72 hr. Thereafter, cell viability was measured and the obtained results demonstrated the ability of Tq@EXOs to effectively decrease the MCF7 viability of IC_10_, IC_25_, and IC_50_ concentrations down to 80%, 69%, and 62%, respectively. However, the outcomes of free Tq were indicative of significantly higher cytotoxicity when compared with Tq@EXOs, which may be due to the controlled and slow release of encapsulated Tq. This result confirmed the capability of Tq@EXOs in inhibiting the growth of MCF7 cells. On the other hand, the lack of any obvious toxicity from Tq@EXOs on L929 as the normal cells at the concentrations of 28 and 56 µM was quite interesting since free Tq exhibited considerable toxic effects under similar conditions. So, this formulation can be a promising attempt for reducing the induced toxicity on normal cells (Figure 7). 

***Cellular uptake by flow cytometry***

To investigate the cellular uptake, Tq@EXOs-FITC and FITC dye (as background control) were applied to MCF-7 cells in order to perform the fluorescent microscopy and flow cytometry analyzes. 

**Figure 8:**
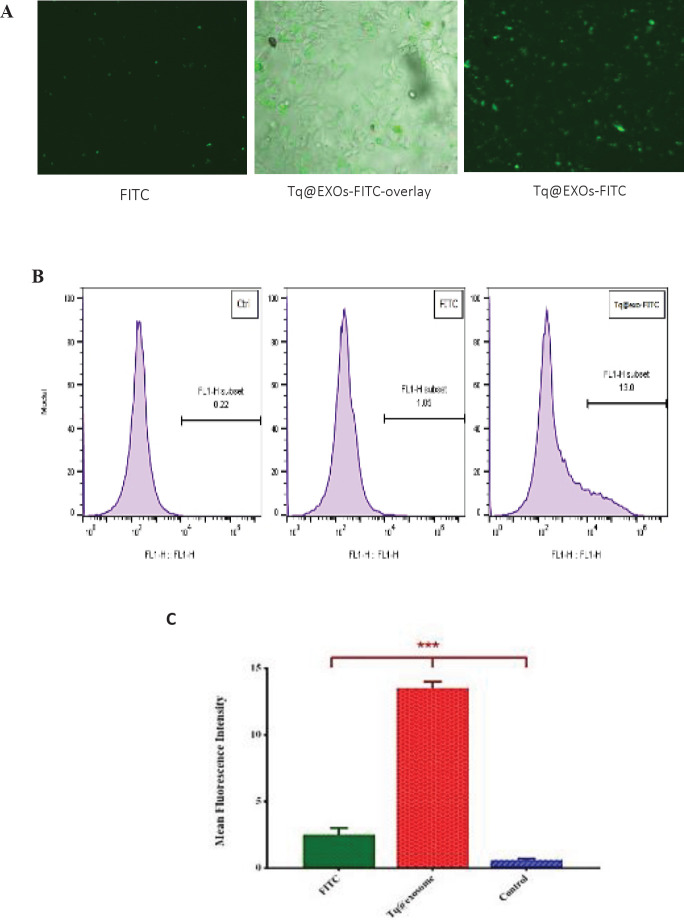
Florescent microscopy and flow cytometry analysis of MCF7 cells upon 4 hr of exposure to Tq@EXOs-FITC and FITC dye. The qualitative analysis of MCF7 cells treated with Tq@EXOs-FITC and FITC dye after 4 hr (A). The quantitative analysis of flow cytometry experiments was also accomplished based on geometric mean florescence intensity of MCF7 cells incubated with either Tq@EXOs-FITC and FITC dye. Data are presented as mean± SD (n = 3) (B and C)

Fluorescent microscopy images displayed the majority of FITC dye at the surface of cells without any signs of uptakes into the cells (Figure 9A). However, the results of fluorescent microscopy and flow cytometry revealed the efficient uptake of Tq@EXOs-FITC by cells throughout a period of 4 hr (Figures 8A and B).

(C) Alizarin Red S staining of the calcium minerals in AdMSCs differentiated into osteocytes. (D) Untreated AdMSCs as the control

**Table 1:** Percentage of encapsulation efficiency of different methods for loading Tq in exosomes

Method	Incubation	Freeze-thawing	Surfactant treatment	Combination
Encapsulation efficiency%	20.8 ± 3.3	16.22 ± 1.4	31.2 + 2.2	57 + 3

## Discussion

Exosomes are defined as natural delivery systems with particular features such as safety, specificity, and stability while exhibiting certain characteristics of the originated cells which provide the delivery of incorporated cargo to specific targets in the body (38). The heterogeneous phospholipid-bilayer and hydrophilic core of these materials can facilitate the loading of both hydrophobic and hydrophilic drugs (1). Exosomes could be secreted and isolated from different types of cells, among which MSCs-derived exosomes were introduced as a suitable carrier for therapeutic agents due to offering desirable properties including cell-specific tropism, proper biocompatibility, and low immunogenicity (39).

Different strategies were applied to perform the loading of therapeutic agents into exosomes including incubation, transfection, physical treatment (sonication, electroporation, extrusion, freeze-thawing, surfactant treatment, and dialysis), and *in situ* assembly & synthesis  (40-42). However, there are several advantages and disadvantages to the usage of these methods. For example, the membrane of exosomes becomes more permeable in the presence of surfactants, which results in the occurrence of more drug loading. In comparison to the simple incubation method, surfactants such as saponin and triton can considerably improve the loading efficiency of different biomolecules into exosomes; nevertheless, it is necessary to watch over the hemolytic activity of saponin throughout the body (43, 44). Moreover, freeze-thaw treatment increases various cargo loading (e.g., drugs, proteins, and peptides) into exosomes through the reconstitution process, however, it provides a less efficient drug loading than that of the sonication and extrusion methods (45, 46). In conformity to reports, sonication causes a reduction in the microviscosity of the exosome membrane, while the extrusion method results in increasing the cytotoxicity and altering the zeta potential of exosomes which may be due to the multiple usage of harsh mechanical forces (46). 

In this study, the loading of Tq into mesenchymal stem cells-derived exosomes was performed through a novel technique (combination of incubation and physical methods including freeze-thawing and surfactant treatment) to enhance encapsulation efficiency and reduce the adverse effects of exerted methods. Although Tq provides the benefits of chemotherapeutic and chemopreventive actions, it is mainly challenged by its high hydrophobicity which leads to low solubility and restricts its bioavailability. 

In this regard, the encapsulation of Tq into exosomes as a natural nanoparticle can improve the obtained instability, drug insolubility, and controlled release while decreasing the induced systemic side effects.

To begin the process, exosomes were isolated from the MScs that were derived from adipose tissue through the application of the ultracentrifugation method to be characterized subsequently. Generally, the exosome membrane is enriched with tetraspanin proteins such as CD9, CD63, and CD81 which are commonly used as exosome markers (47, 48). In this study, the results of western blot analysis confirmed the presence of CD81, CD63, and CD9 in the role of exosomal markers, and also approved the exosome identity by displaying the absence of Bcl2 as the non-exosomal marker. 

Furthermore, FE-SEM analysis was applied to study the morphology of isolated exosomes and the obtained FE-SEM images displayed the homogenous and spherical shapes of nanoparticles. In addition, the results of the DLS analysis are indicative of the obtained mean diameter of 110 nm and a zeta potential value of – 21.3 mV.

The applied loading method can significantly affect the integrity of exosomes, consumption of the loaded drug, and drug loading efficiency. Tian *et al.* reported the loading of doxorubicin into immature dendritic (imDCs) cells’ exosomes with an encapsulation efficiency of up to 20% through performance of the electroporation method (16). As a hydrophobic drug, paclitaxel was encapsulated into exosomes with a maximum of 30% by usage of different loading techniques such as incubation, electroporation, and sonication (17, 49). In this regard, Salarpour *et al*. reported loading of paclitaxel into exosomes in the percentages of about 18.5% and 23% via incubation and sonication procedures, respectively (50). Moreover, Qu *et al.* incubated blood exosomes in the saturated solution of dopamine that contained 0.02% ascorbic acid for a period of 24 hr and achieved an increased encapsulation efficiency of about 16% (51). Provided extracellular vesicles (EVs) from cow milk and Caco-2 cells were loaded with curcumin by being stirred overnight and obtained an encapsulation efficiency of 36% and 12%, respectively (52). In another study, curcumin was incorporated into exosomes by mixing with saponin and incubated at 37 °C, subsequently, the amount of loading was estimated to be 34.46% (53). 

For the very first time, this study attempted to exert the combination of incubation, surfactant treatment, and freeze-thawing and proved the excellence of this approach for increasing the efficiency of drug loading into MSCs-derived exosomes. Our innovative method succeeded in intensifying the encapsulation efficiency up to about 60%, which was superior to the other reported results. 

The MTT outcomes were indicative of Tq@EXOs’ ability to effectively decrease the viability of MCF7. The lower cellular toxicity of Tq@EXOs, when compared with free Tq in MCF-7 cells, could be attributed to the stability of the exosome complex along with sustained and slow release of encapsulated Tq. These results were in agreement with previous studies. The work of Bagheri *et al*. reported the development of MUC1 aptamer decorated exosomes for the delivery of doxorubicin. The determined cellular toxicity of aptamer-targeted DOX-exosomes was observed to be lower than free DOX, which was associated with the structural stability of exosomes and the controlled release of DOX from these formulations (54). 

Furthermore, in our experiment, Tq@EXOs did not cause any obvious cytotoxicity on L929 as the normal cells at concentrations in which free Tq displayed considerable toxic effects. This promising result can be a sign of achieving a reduction in the induced toxicity on normal cells through application of this formulation.

We also examined the uptake of Tq-loaded exosomes into MCF7 cells. According to the results of fluorescent microscopy and flow cytometry, Tq@EXOs-FITC were satisfyingly taken up by the cancer cells. Similar outcomes were reported and confirmed by previous studies. For example, researchers demonstrated that the uptake rate of exosomes by tumor cells was ten times higher than that of liposomes of the same size, which confirmed the excellent specificity of exosomes in targeting cancer cells. Besides, cancerous cells were indicated to internalize exosomes more than normal cells (55, 56).

Considering these facts, our innovative method can effectively provide the achievement of great encapsulation efficiency of thymoquinone to MSCs-derived exosomes and lead to the occurrence of desirable cytotoxicity on cancer cells while reducing the induced toxicity on normal cells. 

## Conclusion

In the present study, we introduced a novel approach by combining three methods of drug encapsulation into exosomes, including simple incubation, surfactant, and freeze-thaw techniques. This approach can serve as an efficient attempt for simultaneous encapsulation of hydrophobic Tq into exosomes. The efficiency of Tq@EXOs was estimated to be about 60% and it is by far one of the best-reported encapsulation percentages when compared with the other available procedures. As the next step, our Tq@EXOs were exerted for cancer therapy against breast cancer cells, preferably as an attempt to solve the challenge of Tq insolubility. The enhanced efficacy was comparable with fewer adverse effects on normal cells.

Our gathered data clearly demonstrated the great potential of exosomes as effective drug carriers for improving anticancer therapies. In the future, this Tq@EXOs formulation can be tested *in vivo* and trialed against different types of cancer cells by application of obtained exosomes from various sources.

## Authors’ Contributions

MH and ZS Processed and collected data and performed experiments; ME Analyzed data and prepared the draft manuscript; ME, LE, and ZS Critically revised the paper; LE and ZS Supervised the research; ME, MH, LE, and ZS approved the final version to be published. 

## Conflicts of Interest

The authors declare that they have no competing interests.
